# Reduced temporal variability of cortical excitation/inhibition ratio in schizophrenia

**DOI:** 10.1038/s41537-025-00568-3

**Published:** 2025-02-18

**Authors:** Frigyes Samuel Racz, Kinga Farkas, Melinda Becske, Hajnalka Molnar, Zsuzsanna Fodor, Peter Mukli, Gabor Csukly

**Affiliations:** 1 Department of Neurology, The University of Texas at Austin, Austin, TX USA; 2 Mulva Clinic for the Neurosciences, The University of Texas at Austin, Austin, TX USA; 3 Department of Physiology, Semmelweis University, Budapest, Hungary; 4 Department of Psychiatry and Psychotherapy, Semmelweis University, Budapest, Hungary; 5 Oklahoma Center for Geroscience and Healthy Brain Aging, University of Oklahoma Health Sciences Center, Oklahoma City, OK USA; 6 Vascular Cognitive Impairment and Neurodegeneration Program, Department of Neurosurgery, University of Oklahoma Health Sciences Center, Oklahoma City, OK USA; 7 International Training Program in Geroscience, Doctoral School of Basic and Translational Medicine/Department of Public Health, Semmelweis University, Budapest, Hungary

**Keywords:** Schizophrenia, Biomarkers, Neural circuits

## Abstract

Altered neural excitation/inhibition (E/I) balance has long been suspected as a potential underlying cause for clinical symptoms in schizophrenia (SZ). Recent methodological advancements linking the spectral slope (*β*) of neurophysiological recordings – such as them electroencephalogram (EEG) – to E/I ratio provided much-needed tools to better understand this plausible relationship. Importantly, most approaches treat E/I ratio as a stationary feature in a single scaling range. On the other hand, previous research indicates that this property might change over time, as well as it can express different characteristics in low- and high-frequency regimes. In line, in this study we analyzed resting-state EEG recordings from 30 patients with SZ and 31 healthy controls (HC) and characterized E/I ratio via *β* separately for low- (1–4 Hz) and high- (20–45 Hz) frequency regimes in a time-resolved manner. Results from this analysis confirmed the bimodal nature of power spectra in both HC and SZ, with steeper spectral slopes in the high- compared to low-frequency ranges. We did not observe any between-group differences in stationary (i.e., time-averaged) neural signatures, however, the temporal variance of *β* in the 20–45 Hz regime was significantly reduced in SZ patients when compared to HC, predominantly over regions corresponding to the dorsal attention network. Furthermore, this alteration was found correlated to positive clinical symptom scores. Our study indicates that altered E/I ratio dynamics are a characteristic trait of SZ that reflect pathophysiological processes involving the parietal and occipital cortices, potentially responsible for some of the clinical features of the disorder.

## Introduction

Schizophrenia (SZ) is one of the most common psychiatric disorders with a lifetime prevalence of about 1%^1^ and an increasing trend of incidence over the past 30 years^2^. The precise pathophysiology of SZ is yet unknown and thus SZ management (including diagnosis) mostly relies on assessment of clinical presentation^3,4^. The vast phenotypic heterogeneity^5^ observed in SZ, however, renders it immensely difficult to untangle the possible etiologies behind its various symptoms, and thus research focusing on identifying biomarkers that might help in better understanding its multifactorial pathomechanisms, progression monitoring or prognosis estimation is of central importance.

Power spectral abnormalities in the electroencephalogram (EEG) are extensively studied in SZ^6^; however, traditional research mostly focused on deviations in band limited power (BLP). Typically, BLP analyses assess neural activity in characteristic frequency regimes (e.g., 8–13 Hz for *alpha*) by integrating spectral power in the given regime. These approaches, however, most often disregard that neural power spectra are arguably composed of broadband fractal ($1/{f}^{\beta }$) ‘background activity’ with superimposed oscillatory peaks, and these components might represent different neural processes^7,8^ and thus should be treated separately. Indeed, separating fractal and oscillatory components of EEG power spectra appears to be relevant in SZ, too^9^. Furthermore, it has been proposed lately that the spectral slope (*β*) of the broadband component—i.e., the slope of the linear regression line fitted on log-transformed power over log-transformed frequency—can characterize the proportion of incoming excitatory and inhibitory activity, with flatter spectra indicating higher E/I ratio and vice versa^10^. This advancement might prove to be immensely relevant in SZ research, as altered excitatory NMDA^11,12^ and inhibitory GABA^13,14^ brain signaling has been consistently reported in SZ (for recent reviews, see e.g., refs. ^15,16^). Accordingly, abnormal excitation/inhibition (E/I) balance has been proposed as one of the underlying mechanisms of affected perceptual and cognitive functions in SZ^17–20^. Supporting this theory, by utilizing the concept outlined in ref. ^10^ Molina et al. ^21^ analyzed EEG recordings of SZ patients and revealed stronger inhibitory tone (i.e., steeper spectra) in contrast to healthy controls (HC), which was also correlated to attention and vigilance in a memantine stress test. Increased *β* was also identified in SZ during a self-referential task, and it was also found associated with negative symptom scores^22^.

Even though *β* is a promising novel biomarker in SZ given the apparent presence of aberrant E/I signaling, previous findings in the literature warrant further considerations. It has been shown that the power spectra of electrophysiological recordings can be characterized with different slopes in various frequency regimes, i.e., *β* can change with the frequency band^8,23–25^. This phenomenon is often referred to as bi- or multimodality^9,24,26,27^. Moreover, even though the relationship between E/I ratio and *β* proposed by Gao et al.^10^ was supported by multiple independent studies^28,29^, literature also indicates that this relationship is reversed for slow (<5 Hz) brain rhythms^30,31^. This aspect might be especially important in SZ; while in^21^ a single slope estimate was obtained from the 4–50 Hz domain, it was also shown recently that EEG spectra are bimodal in SZ with a flatter low-range and steeper high-range domain^9^. Finally, previous evidence suggests that neural activity can express multifractal characteristics in healthy population^32^ as well as in SZ^33^. The presence of multifractality indicates that the fractal scaling exponent of the dynamic process—that is equivalent to its spectral slope *β*^34,35^ – changes over time^36^. Accordingly, a single, global estimate might be insufficient to properly characterize the dynamics of neural E/I ratio in SZ and instead a time-resolved analysis characterizing both the mean and variance of *β* over time appears as a more reasonable approach.

Disentangling how E/I balance is affected and plausibly related to clinical symptoms in SZ is crucial for better understanding its pathophysiology and thus could open new venues in its diagnosis and management. Therefore, in line with the aforementioned notions, we analyzed EEG recordings of 30 patients with SZ and contrasted findings with those from 31 HC individuals in this cross-sectional, case-controlled study. We hypothesized that the power spectra of EEG recordings in SZ patients will be (i) bimodal and thus characterized with different scaling exponents in low- and high-frequency regimes, and that (ii) this property fluctuates over short time scales. We also expected to find (iii) steeper spectra (within-subject mean in time, *μ*) and altered temporal variability (within-subject standard deviation in time, *σ*) of *β* in SZ when compared to HC, indicating disrupted E/I balance. Finally, although there is indication on the role of E/I balance in SZ pathology, there is no clear consensus yet on how E/I ratio as captured via *β* might relate to the clinical appearance of SZ. Our goal was therefore also to (iv) gain novel insights on this aspect.

## Materials and methods

### Participants and clinical measures

A total of 38 patients with SZ and 39 HC individuals were enrolled in this study, of which 8-8 individuals were excluded from analysis due to low data quality (see below). Demographic and clinical information of the final study sample are presented in Table 1. There were no statistical differences between the two groups in terms of age, proportion of female/male individuals or years spent in education. The study was reviewed and approved by the Semmelweis University Regional and Institutional Committee of Science and Research Ethics (registration number: 197/2015, date: 10/05/2015). All participants provided written informed consent before enrollment in line with the Declaration of Helsinki.

**Table 1:** Demographic information of study groups with clinical characteristics medication data for the schizophrenia group.

Variable	SZ (*n* = 30)	HC (*n* = 31)	Statistics	*p*-value
Sex	11F/19M	13F/18M	${\chi }^{2}$ = 0.1773	*p* = 0.6737
Age	33.07 ± 9.73	33.06 ± 10.31	t = −0.0008	*p* = 0.9993
Years in education	16 [8; 18]	16 [12; 18]	z = 1.0439	*p* = 0.2965
Illness duration	8.17 ± 8.57			
CPZ equivalent dose	394.16 ± 346.01			
PANSS total score	60.63 ± 17.74			
PANSS general subscore	30.10 ± 9.05			
PANSS negative subscore	15.90 ± 5.70			
PANSS positive subscore	14.63 ± 5.12			
CPZ equivalent dose	394.16 ± 346.01			

Medication information (SZ group)				
Medication name	*n* (mean dose ± SD mg)		Medication type	
Quetiapine	3 (500 ± 285 mg)		antipsychotics	
Risperidone	1 (2 mg)			
Flupentixol decanoate	1 (20 mg)			
Clozapine	13 (135 ± 132 mg)			
Haloperidol	3 (3.75 ± 1.1 mg)			
Olanzapine	4 (8.125 ± 2.4 mg)			
Amisulpride	5 (430 ± 338 mg)			
Aripiprazole	8 (16.875 ± 5.3 mg)			
Lithium	5 (850 ± 224 mg)		Mood stabilizers	
Valproate	2 (950 ± 71 mg)			
Lamotrigine	1 (200 mg)			
Clonazepam	9 (1.5 ± 0.9 mg)		Benzodiazepines	
Alprazolam	1 (0.5 mg)			
Paroxetine	1 (20 mg)		Antidepressants	
Sertraline	1 (50 mg)			
Citalopram	1 (20 mg)			

*SZ* schizophrenia, *HC* healthy control, *F* female, *M* make, *CPZ* chlorpromazine equivalent dose, *PANSS* positive and negative syndrome scale, *SD* standard deviation.

Patients with SZ were recruited from the Department of Psychiatry and Psychotherapy (DPP), Semmelweis University, Budapest, Hungary, while HC participants were recruited from the general population of Budapest, Hungary. All patients met the criteria for SZ based on the Structured Clinical Interview for Diagnostic and Statistical Manual of Mental Disorders, 5th Edition (DSM-5)^4^. Psychiatric symptoms on the Positive and Negative Syndrome Scale (PANSS)^37^ were evaluated by trained psychiatrists. All data collection were carried out at the DPP between May 2016 and July 2018. Patients did not go through a medication washout period at the time of recordings and PANSS evaluation; detailed information on medication is shown in Table 1. No follow-up data was analyzed in this study.

Selection criteria for all participants included the following: no history of stroke, intellectual disability, epileptic seizure, substance dependence or abuse in the past three months, and no history of head injury with loss of consciousness for more than ten minutes. Furthermore, an exclusion criterion for patients was presence of any other mental disorder (according to DSM-5), and for healthy controls, presence of any mental disorder.

### EEG recording, quality control and pre-processing

EEG in both cohorts was collected at 1000 Hz sampling rate using a 64-channel Neuroscan amplifier from standard 10-10 locations referenced to linked mastoids (M1/M2). Measurements were carried out in a dimly lit, sound-attenuated room. Participants were seated in an armchair and were instructed to remain idle but awake (resting state) with their eyes closed (EC) for two minutes. After collection, raw EEG recordings were visually inspected by two investigators independently. During the screening procedure, investigators were unaware of the data group label (HC or SZ) and they only contrasted outcomes once all steps were completed. Channels identified as noisy/bad (e.g., amplitude values consistently spanning outside ±100 µV range, flatline signal, aberrant power spectrum) in over 10 participants (channels AF7, AF8, AFz, F1, F2, TP7, TP8), as well as M1/M2 electrodes were removed from all recordings. Artifact free segments i.e., those absent of overt spikes or distributed high frequency or large amplitude noise were isolated in all recordings if they were marked as ‘clean’ by both experimenters independently, and a 30-s-long, continuous epoch from these was selected randomly for further analysis. This epoch length was decided upon as a reasonable compromise among the study sample, as being long enough for meaningful analysis of dynamics, yet it could be obtained in most study participants. In turn, those participants where such a segment could not be identified were excluded from further analysis, yielding a final sample of 31 HC and 30 SZ participants, with 55 channels each.

Data was preprocessed using Matlab (MathWorks, Nattick, MA) and the EEGLAB toolbox^38^. EEG was first downsampled to 256 Hz, then bandpass filtered between 0.5 and 100 Hz using a 4th order zero-phase Butterworth filter, with additional notch filters at 50, 100 and 150 Hz. Components of non-neural origin—such as muscle and heart activity—were removed using independent component analysis and the multiple artefact rejection algorithm (MARA) technique^39,40^ with default parameter settings. MARA is an automated, machine learning-based software plugin to EEGLAB that classifies independent components as artifacts (e.g., line noise, muscle activity or eye movements) or neural activity based on six temporal, spectral or spatial features^41^ and is widely utilized in the scientific literature. For more details on MARA, the reader is referred to the original publications^39,40^ or^41^. In the total study cohort, the average number of removed components was 26.55 ± 10.16 (~48.27%), while 24.94 ± 10.78 for the HC and 28.37 ± 9.25 for the SZ groups individually. These proportions of neuronal vs. artifactual components are in line with those expected^39,40^. There was no statistical difference between the number of rejected components between the HC and SZ groups (*p* = 0.1804, two-sample *t-*test). Finally, data was re-referenced to the common average reference.

### Data analysis

**Fig. 1:**
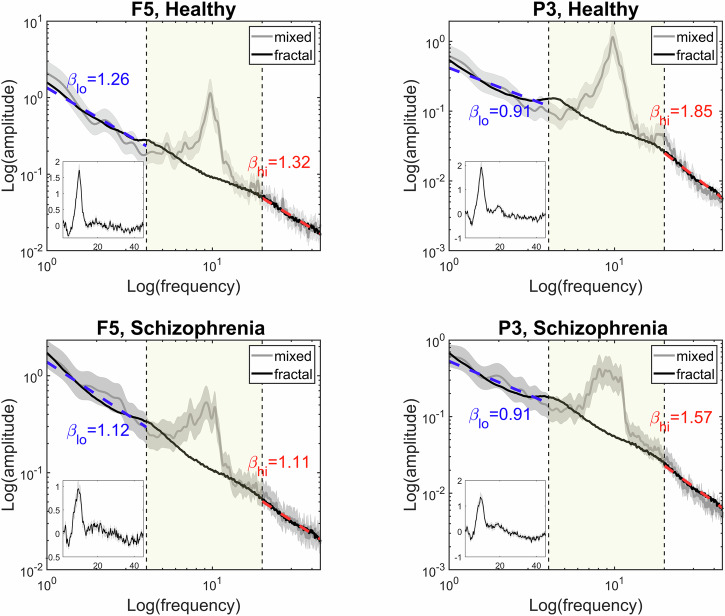
Illustration of group-averaged power spectra from two representative cortical regions F5 (left) and P3 (right) in the HC (upper panels) and SZ (bottom panels) cohorts. In every panel, gray lines indicate the mixed (i.e., raw), while black lines the isolated fractal power spectra, respectively, with gray shaded areas denoting the standard error of the mean. Inset plots show corresponding isolated oscillatory spectra, obtained by subtracting the fractal from the raw (mixed) spectra. Boundaries of the two frequency regimes of interest (1–4 Hz and 20–45 Hz) are indicated with black dashed lines, while the range excluded from slope estimation (4–20 Hz) is marked in yellow. Blue and red dashed lines are illustrative and depict the power-law functions fitted on the fractal spectra in the low- and high-frequency regimes, respectively, with their corresponding slopes presented in matching colors. The panels illustrate that IRASA is effective in separating the fractal from the mixed power spectrum with minimal residual effect, and the utilized parameter settings and employed frequency regimes allow for unbiased estimates. In both groups low- and high-range slopes are roughly equal over F5 region, while the spectrum appears steeper in high- compared to low-range over P3. HC healthy control, SZ schizophrenia, IRASA irregular resampling auto-spectral analysis.

For characterizing neural E/I ratio, the spectral slopes were estimated using the irregular-resampling auto-spectral analysis (IRASA) technique^42^. IRASA decomposes the power spectrum into broadband fractal and superimposed oscillatory components, so that *β* can be estimated from the fractal component without the biasing effects of oscillatory (such as alpha) peaks^42^. It is important to note that despite the spectral slope being negative ($1/{f}^{\beta }$), according to convention^43^ we will use its absolute value throughout this manuscript, and therefore an increase in *β* will denote a steeper spectrum, and vice versa. In this work we employed a bimodal analysis approach, in that spectral slopes were estimated separately for low- and high-frequency regimes (see below); the process is illustrated on Fig. 1 from two representative regions in the HC (upper panels) and SZ (lower panels) groups. For details of the IRASA method and its theoretical background, please see the Supplementary Material and the original publications^42,44^.

In the time-resolved analysis, the 30-s EEG segments were first divided into 8-second-long epochs with 75% overlap (yielding 12 windows). The window size was defined following previous recommendations as at this time scale EEG still can be treated as stationary yet providing ideal frequency resolution^6^, while a 75% window overlap—i.e., 25% step size – in dynamic fractal analysis has been proven effective in picking up changes in fractal scaling exponent despite introducing redundancy^45,46^. Then, IRASA was applied to each 8-s epoch to reconstruct the spectrum and separate fractal and oscillatory spectral components in the 1–45 Hz regime for all channels. Frequency resolution was set to 0.0625 Hz, and the rescaling factor *h* ranged from 1.1 to 2.6 in 0.1 increments, to avoid potential ‘smearing’ effects around the alpha peak (see Supplementary Material in ref. ^9^). Spectral slopes were estimated in the 1–4 Hz (${\beta }_{{lo}}$) and 20–45 Hz (${\beta }_{{hi}}$) ranges independently via ordinary least squares (OLS) linear regression of log-transformed fractal power on log-transformed frequency (see Fig. 1). These frequency regimes were selected in line with previous studies^29,30^ and considering the filtering effects introduced by up- and downsampling during IRASA analysis (see^9^ and its Supplementary Material). For control purposes, slope estimates were obtained from the broadband 1–45 Hz spectra, too (${\beta }_{{bb}}$).

**Fig. 2:**
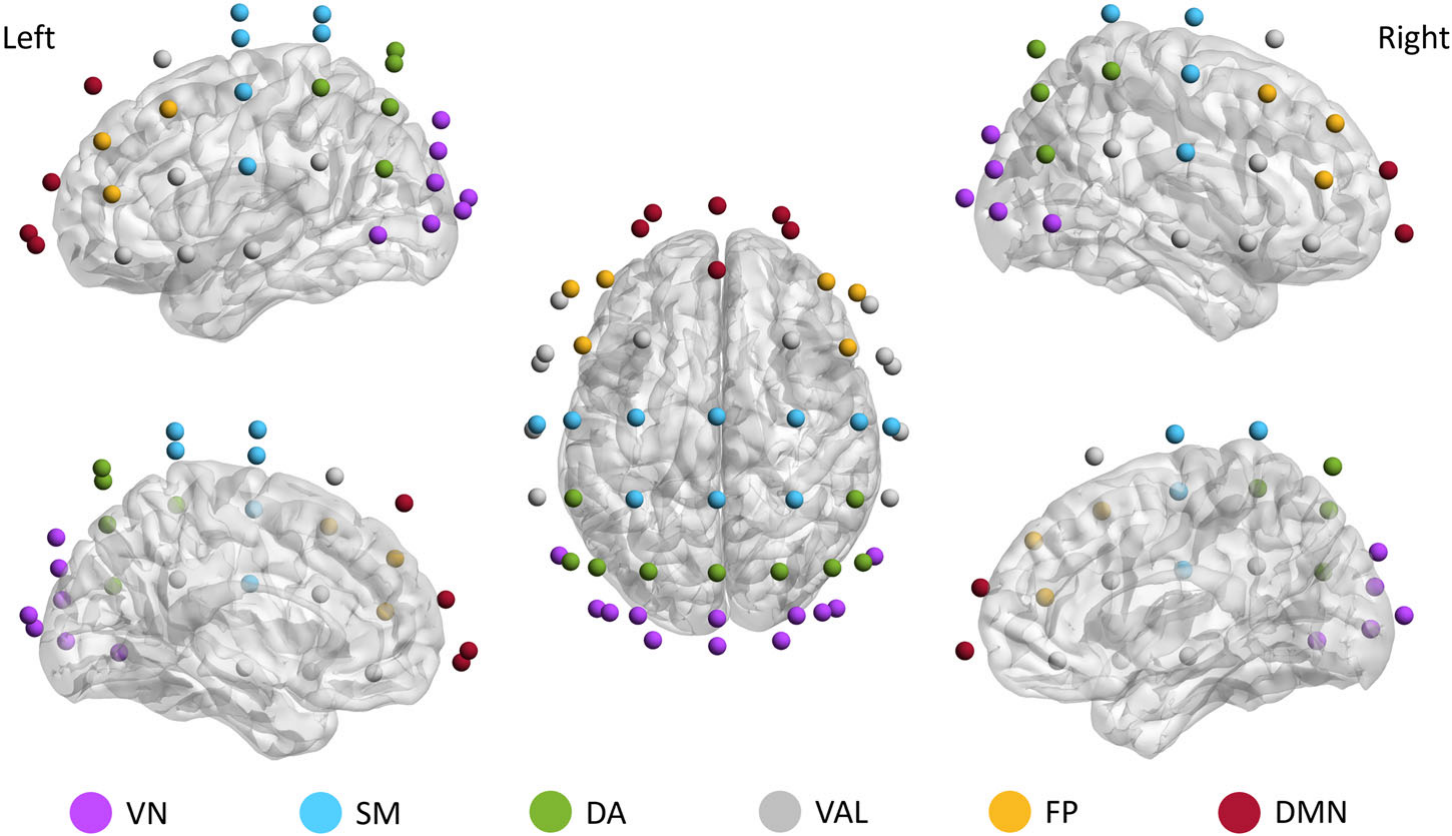
Electrode montage and resting-state network (RSN) association. The six RSNs include the visual network (VN, 12 channels), the somatomotor network (SM, 10 channels), the dorsal attention network (DA, 9 channels), the combined ventral attention- and limbic networks (VAL, 12 channels), the frontoparietal network (6 channels) and the default mode network (6 channels). Channels correspondence to different RSNs are indicated with various colors (VN: purple; SM: blue; DA: green; VAL: gray; FP: orange and DMN: red).

To reduce dimensionality and improve physiological interpretability, channels were grouped according to established resting-state networks (RSNs) of the brain^47^. In line with our previous approaches^9,48^, channels were assigned to the RSN whose activity they most likely register based on the work of Giacometti and colleagues^49^. Out of the 7 RSNs proposed by Yeo and coworkers^47^, we isolated the visual network (VN), the somatomotor network (SM), the dorsal attention network (DA), the frontoparietal network (FP) and the default mode network (DMN), while channels corresponding to the ventral attention and limbic networks were pooled (VAL) due to limitations in spatial resolution. The 55-channel montage and RSN-channel assignment is illustrated on Fig. 2 using the BrainNet Viewer software^50^; spectral slope estimates were averaged over channels belonging to RSNs for each time window. Finally, for every participant, neural indices (RSN-wise) were characterized by their mean and standard deviation over time. These will be indicated by $\mu (\cdot )$ and $\sigma (\cdot )$, respectively, e.g., the temporal standard deviation of high-range spectral slope will be denoted as $\sigma ({\beta }_{{hi}})$.

### Statistical analysis and relationships with clinical scores

The obtained neurophysiological indices were first tested for normality using the Lilliefors test, then between group differences were assessed via two-sample *t-*tests in case of normal distributions, while Mann-Whitney U tests otherwise. The bimodal nature of the spectra was assessed in the SZ and HC groups independently by contrasting $\mu ({\beta }_{{lo}})$ and $\mu ({\beta }_{{hi}})$ values with paired *t-*tests or Wilcoxon signed rank tests, depending on data normality. Note that this analysis was carried out for each channel individually. For all variables, outcomes were adjusted for multiple comparisons using the false discovery rate (FDR) method of Benjamini and Hochberg^51^ at a statistical significance level of 0.05. Finally, neural indices of the SZ group that indicated significant between-group difference were contrasted with general (GEN), negative (NEG), positive (POS) and summed (SUM) PANSS symptom scores. In that, first the confounding effects of age, sex, years in education, disease duration and CPZ were regressed out from all variables using multiple linear regression. Then, the Pearson or Spearman correlation coefficient (depending on data normality) was computed between the neural indices and symptom scores. Notably, correlational analyses were performed only for those neural indices where significant between-group difference was found. Given the exploratory nature of this analysis, outcomes were not adjusted for multiple comparisons.

## Results

### Bimodality of power spectra

**Fig. 3:**
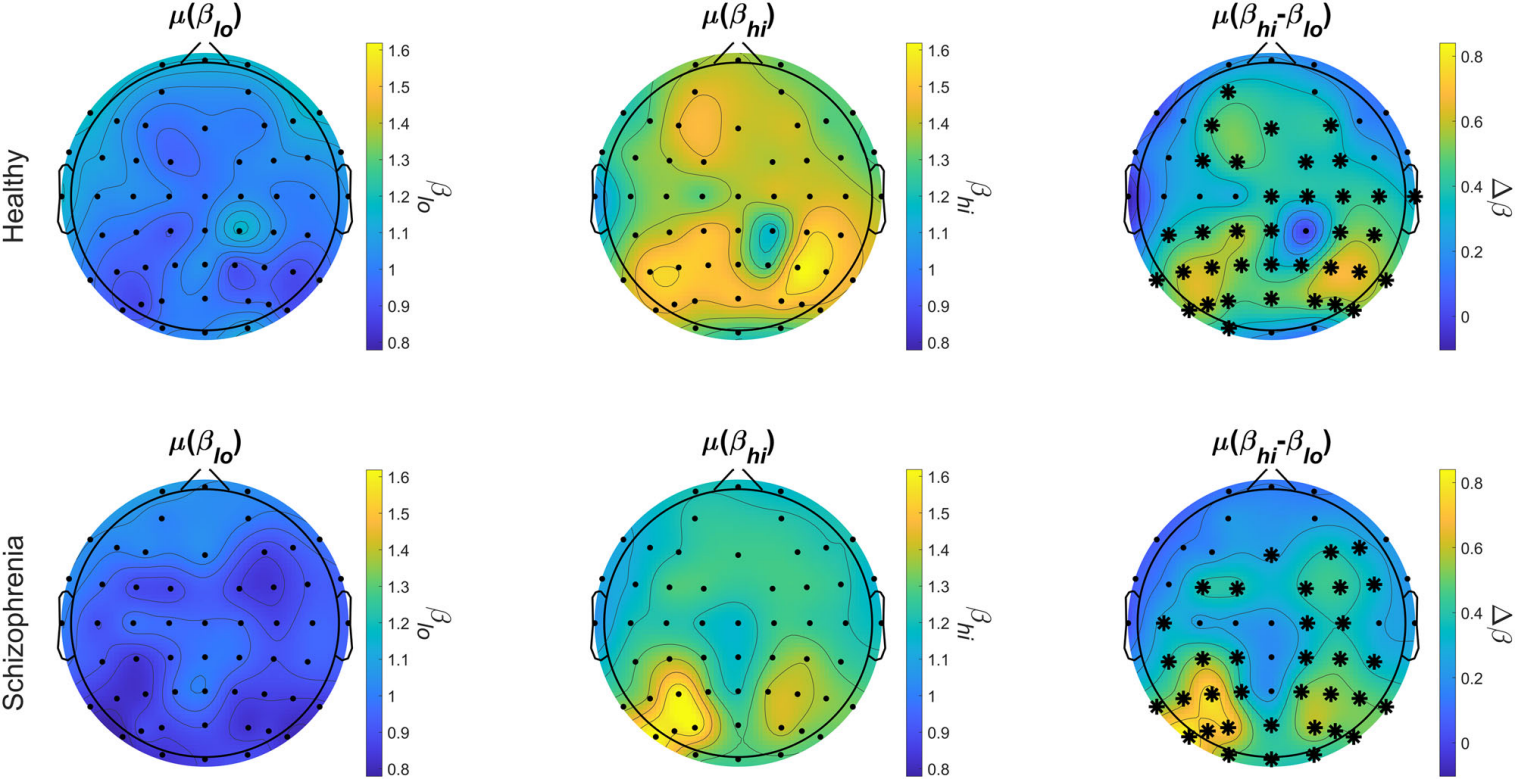
Group average differences between low- and high-range spectral slopes in healthy control (upper row) and schizophrenia (lower row). Lower $\mu ({\beta }_{{lo}})$ values (left column) compared to $\mu ({\beta }_{{hi}})$ (middle column) are apparent in both study groups. The right column shows the difference ($\mu \left({\beta }_{{hi}}\right)-\mu ({\beta }_{{lo}})$, with asterisk symbols denoting statistically significant bimodality (*p* < 0.05, adjusted) over most of the cortex with a parieto-occipital dominance.

In both study groups, EEG spectra were steeper in the high- compared to the low-frequency regime over the majority of channels (Fig. 3). Precisely, ${\mu (\beta }_{{hi}})$ was higher than $\mu ({\beta }_{{lo}})$ over 36 and 35 out of 55 cortical locations in the HC and SZ groups, respectively. This bimodality could be observed most prominently over parieto-occipital and central regions, while EEG spectra over temporal-frontal brain regions were found rather unimodal (Fig. 3, right). These results – in accordance with^9^—confirm that EEG spectra should be treated as bimodal in the 1–45 Hz range with steeper slopes characteristic of high- compared to low-frequency regimes, and this property appears to be mostly preserved in SZ when compared to HC.

### Reduced temporal variance of high-range spectral slope in Schizophrenia

**Fig. 4:**
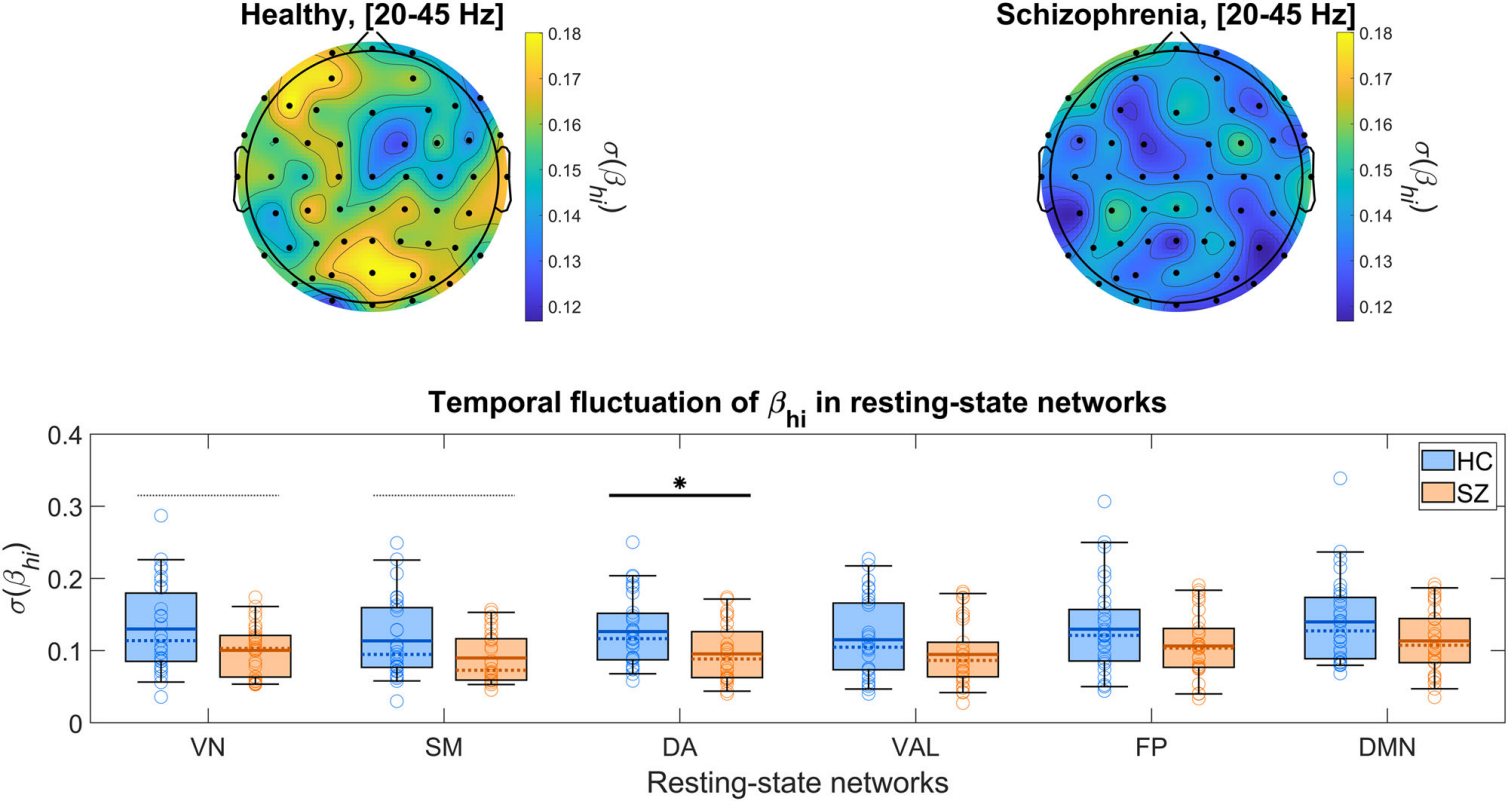
Temporal variability of *β*_*hi*_ in the healthy (HC) and schizophrenia (SZ) groups. The upper left and right panels show the topology of $\sigma ({\beta }_{{hi}})$ in HC and SZ, respectively. The lower panel shows resting-state network (RSN)-wise analysis of $\sigma ({\beta }_{{hi}})$, with black, straight horizontal line and asterisk indicating significant between-group difference (p < 0.05, adjusted). In all box plots, continuous and dotted lines indicate the mean and median, respectively, the rectangular area denotes the inter-quartile range, horizontal whiskers indicate 5th and 95th percentile, and circles show the individual samples. Note that black, horizontal dotted lines indicate between group differences that were rendered non-significant by multiple comparisons adjustment. ${\beta }_{{hi}}$: high-range (20–45 Hz) spectral exponent; $\sigma ({\beta }_{{hi}})$: standard deviation of ${\beta }_{{hi}}$ over time.

We did not find any differences between HC and SZ in the mean spectral slopes in either frequency regimes following FDR-adjustment (see also Fig. 3). However, in terms of temporal variability, $\sigma ({\beta }_{{hi}})$ was found generally reduced in the SZ population (Fig. 4). This difference was most apparent over the parieto-occipital cortex, with another cluster over the left frontal lobe. RSN-wise statistical analysis confirmed significantly lower $\sigma ({\beta }_{{hi}})$ in SZ in the DA network (mean 0.1264 vs. 0.0955, *t*_*59*_ = 2.7353, *p* = 0.0082), while this difference was exhibited as a tendency for VN (mean 0.1299 vs. 0.1004, *t*_*59*_ = 2.3317, *p* = 0.0232, non-significant after FDR-adjustment) and SM(median 0.0947 vs. 0.0728, *Z* = 2.0557, *p* = 0.0398, non-significant after FDR-adjustment). The same pattern was also exhibited by the rest of the RSNs, namely, VAL (median 0.1047 vs. 0.0864, *Z* = 1.5797, *p* = 0.1142, non-significant), FP (mean 0.1297 vs. 0.1064, *t*_*59*_ = 1.6672, *p* = 0.1008, non-significant) and DMN (mean 0.1398 vs. 0.1134, *t*_*59*_ = 1.9373, *p* = 0.0575, non-significant), although not reaching statistical significance. In comparison, no significant between-group differences were identified for ${\beta }_{{lo}}$ either in terms of mean or temporal variability (see Supplementary Fig. S1 for the latter). Note that also no significant between-group differences were found for ${\beta }_{{bb}}$ at all. Accordingly, these results imply that E/I ratio has similar baseline level in SZ, while its temporal variability is reduced compared to what is observed in the HC population.

### Correlations with clinical symptom scores

**Fig. 5:**
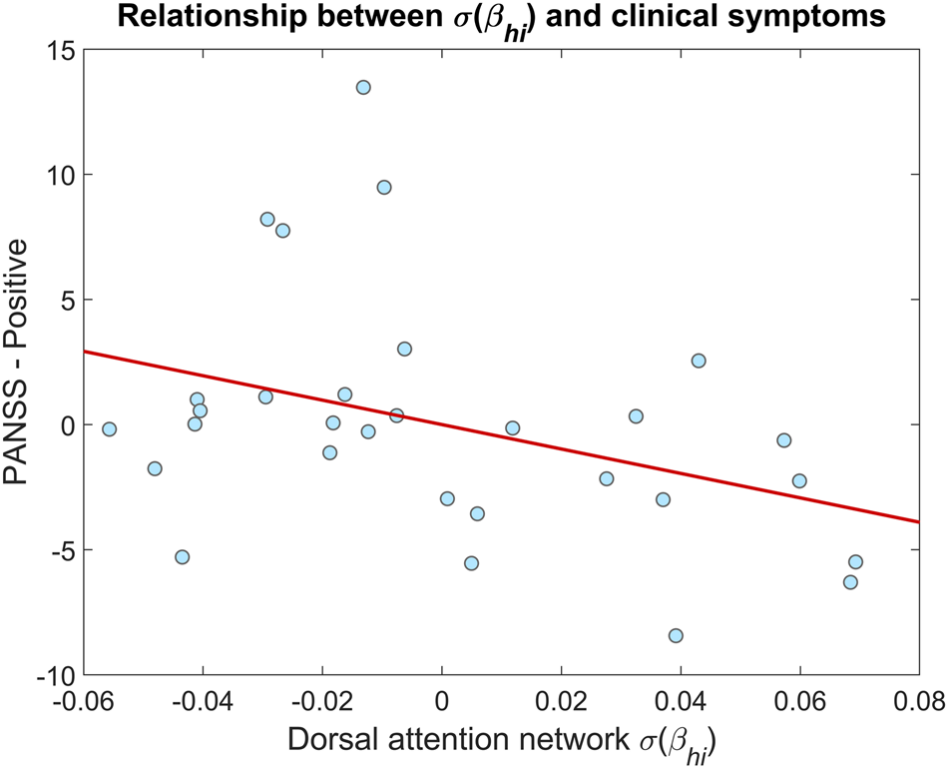
Relationship between *σ*(*β*_*hi*_) over the dorsal attention network and positive PANSS scores. Both variables were adjusted for the plausible confounding effects of age, sex, years of education, disease duration and chlorpromazine daily equivalent dose. The straight red line denotes the ordinary least squares regression line, which only serves illustrative purposes. $\sigma ({\beta }_{{hi}})$: standard deviation of high-range (20–45 Hz) spectral exponent over time; PANSS positive and negative syndrome scale.

Finally, we computed if the observed between-group difference—in $\sigma ({\beta }_{{hi}})$ over the DA network – were associated with PANSS scores in the SZ group. An anticorrelated relationship between $\sigma ({\beta }_{{hi}})$ of DA and positive PANSS scores could be identified; however, assumptions of OLS regression were not satisfied (normality of the residuals in particular) and thus we characterized this relationship via Spearman correlation coefficient (*r* = –0.3989, *p* = 0.0298) after adjusting for confounding variables (Fig. 5).

## Discussion

Neural markers of SZ derived from EEG data have been a subject of research interest for decades^6,52^. Previous analyses revealed mostly consistent abnormalities in SZ in terms of resting-state EEG, such as increased *delta* (1–4 Hz) and/or *theta* (4–7 Hz) BLP over frontal regions^53,54^, globally reduced *alpha* (8–13 Hz) BLP^53^ or abnormalities in *gamma* (30–80 Hz) synchronization^55^. However, most of these efforts did not take into consideration of the broadband spectral characteristics and $1/{f}^{\beta }$ nature of neural activity. Our presented results provide important novel insights on these aspects.

Altered neural E/I ratio has long been hypothesized as one of the key etiological factors of SZ^3,15^, supported by both molecular^12,56^, electrophysiological^21^ or transcranial magnetic stimulation^20^ evidence. The proposal of *β* as a non-invasive marker for E/I ratio^10^ and simultaneously developed novel analysis strategies^42,57,58^ led to a rapid upsurge of interest in E/I phenomena recently; however, such studies investigating SZ are yet scarce^21,59^. Our goal was to address this knowledge gap with notable key considerations in mind. The hypothesis that an increased (decreased) E/I ratio results in the reduction (increase) of *β* has been confirmed empirically by multiple independent studies using pharmaceutical interventions^21,29,31^, optogenetic manipulation^28^, computer simulations^60^ or a priori hypotheses based on histological structure^10^. Nonetheless, while the model of Gao and coworkers indicated that this result holds mainly in the ~20–50 Hz frequency regime, the range of estimation in practice varies broadly among studies. This may be concerning according to the results of Becker and colleagues^30^, which implied that the relationship between *β* and inhibitory tone is reversed in the low-frequency (<5 Hz) regime. Even though that study used spontaneous resting *alpha* BLP as a proxy for inhibition^61,62^, Muthukumarshawamy and Lilley^31^ showed that increased GABA-ergic tone via administering the GABA reuptake inhibitor Tiagabine resulted in simultaneous decrease in low- (<2.5 Hz) and increase in high-range (>20 Hz) spectral slopes. This taken together with previous reports on bimodal nature of EEG spectra^8,9,23,24^ warrants the low- and high-frequency regimes of the power spectrum to be treated separately. Our results support this notion: the bimodal analysis not only confirmed significant difference of ${\beta }_{{lo}}$ and ${\beta }_{{hi}}$ but also indicated that dynamics governing only the latter appear to be affected in SZ. In contrast, when we characterized spectra with a single broadband slope estimate, no group-level differences were identified. We also did not observe the steeper grand-averaged EEG spectrum in SZ as reported in ref. ^21^; however, in that study the authors analyzed data collected during an auditory oddball paradigm and slopes were estimated from the 4–50 Hz range, which might explain this discrepancy^63^.

In accordance with our previous findings^9^ on an independent HC and SZ-patient cohort^64^, the extent of bimodality was stronger over parieto-occipital regions, while less prominent or non-existent over frontal areas. As these regions are also normally expected to exhibit stronger resting alpha power in eyes-closed state^65^, this might raise the possibility that the presence of a prominent alpha peak confounded the results via spectral leakage. Our analysis pipeline was designed to counter this possibility in IRASA employing Hanning windowing to reduce leakage as well as we increased the set of rescaling factors to 1.1:0.1:2.6 from the recommended standard 1.1:0.05:1.9 range^42^. While Fig. 1 indicates that with these parameter settings the isolated fractal spectra were not compromised in the employed frequency regimes, we cannot rule out the possibility of an interplay between resting alpha power and the slope of the fractal spectrum. In fact, it is important to emphasize that such a pattern is likely to emerge along physiological—instead of methodological – principles. Namely, it is argued that alpha power is a proxy for inhibitory tone^61,62^, which also served as the theoretical foundation for the analysis conducted by Becker and colleagues^30^. In this context, larger alpha power and steeper slope in the higher frequency regime would confluently indicate stronger inhibitory tone, and therefore our findings regarding $\sigma ({\beta }_{{hi}})$ coinciding with areas with more prominent alpha activity might very well reflect the same neural phenomenon through different means. A more elaborate investigation of this question is possible by a detailed analysis of ‘traditional’ spectral measures (such as BLP), which we believe is beyond the scope of this paper; however, our plan is to publish those outcomes shortly in a companion article.

The lack of between-group difference in $\mu ({\beta }_{{lo}})$ and $\mu ({\beta }_{{hi}})$ values implies that overall E/I balance is maintained in SZ on longer time scales. As noted, this might appear to be in contrast with previous, similar literature^21,22^; however, these studies analyzed EEG collected during task performance and not in resting-state. Indeed, our previous bimodal analysis of an independent resting-state EEG dataset also indicated no difference in $\mu ({\beta }_{{lo}})$ and $\mu ({\beta }_{{hi}})$ between HC and SZ groups^9^. Our central result presented here shows that this breaks down on shorter time scales: SZ patients expressed reduced temporal fluctuations in ${\beta }_{{hi}}$ when compared to HC individuals. Precisely, reduced $\sigma ({\beta }_{{hi}})$ was most prominent over the parieto-occipital cortices (see Fig. 4), regions likely representing the activity of the DA network^49^. Even though SZ-related EEG abnormalities are more commonly found over the frontal and temporal lobes^52^, the importance of the parietal cortex regarding attention, working memory and other cognitive domain deficits in SZ has been emphasized^66,67^. Our results are in line with the latter notion, especially with^21^ linking E/I ratio to attentional and vigilance deficits in SZ. Therefore, investigating variability of EEG spectral power and E/I ratio measures in attentional and working memory tasks in SZ appears as an important future research direction, for which our presented analytical approach could prove valuable. Additionally, reduced $\sigma ({\beta }_{{hi}})$ may also imply loss of multifractality in neural dynamics. Multifractality often emerges in physiological systems as a result of antagonistic feedback mechanisms^68^ and its loss can indicate the involvement of either, or both components^36,69^. In terms of neural circuits, this might reflect the disruption of large-scale excitatory-inhibitory feedback loops^70^, which can be expected in SZ. Better understanding of this phenomenon can bring novel insights on the pathophysiology of SZ and its symptoms, and therefore it is one of our key goals in the future.

Our analysis revealed an association between the investigated neural indices and clinical symptoms; however, given the exploratory nature of these results—as outcomes were not adjusted for multiple comparisons—we must exercise caution regarding their interpretation. Precisely, E/I dynamics were negatively correlated with positive PANSS scores in the DA network (Fig. 5). The pathophysiology of positive symptoms in SZ is not yet fully understood and thus is a subject of intense research^71,72^. As indicated previously, the parietal cortex was rather associated with cognitive deficits in SZ^66,67^, in line with the plausible affectedness of the DA network. Neural E/I ratio have been linked to multiple cognitive domains recently in various conditions such as aging or multiple sclerosis^29,73–75^, which tentatively projects that *β*-based E/I analysis could be helpful in better understanding its cognitive disfunction in SZ. On the other hand, positive symptoms can be well-managed with antidopaminergic medication in roughly two-thirds of cases^76^, which raises the possibility that ${\beta }_{{hi}}$ fluctuations in the DA network might be related to the activity of dopaminergic system. On the other hand, it is important to note that patients were on medication during the time of the assessments, and therefore it is possible that outcomes are confounded by the variability in therapy response to different pharmaceuticals. While we cannot confirm or rule out this possibility based on the current study sample, this is an important future direction that might yield valuable novel insights on the neurobiology of SZ, but also on identifying neural features that can be utilized to estimate therapy response. Nevertheless, our current dataset and experimental design could not allow us to provide further explanations for these associations, however, we consider it as an important direction to pursue in the future, such as investigating how the spectral slope is modulated by antipsychotic medication^77^ or how it is related to performance in cognitive tasks requiring spatial attention and goal-directed behavior^78^ in patients with SZ.

While our study provides novel insights into affected neural dynamics in SZ, it also has certain limitations. Analyses were performed on resting-state data, and thus the relevance of the identified alterations in terms of cognitive deficits could not be investigated and requires future research. This issue is even more relevant in light of recent reports suggesting that the mean spectral slope might indicate alterations in task-response rather than resting activity in SZ^21,22^. It is important to note that our characterization of EEG spectral dynamics is likely incomplete given that it was obtained from 30 s of continuous activity. This was a technical limitation of our dataset, as longer continuous segments could only have been obtained from a sufficiently smaller number of participants, thus reducing statistical robustness. On the other hand, the known scale invariant property of neural activity suggests relevant dynamic patterns on time scales beyond 30 s^27,33^, which warrants further research. Also, EEG data was evaluated in the sensor space and thus we cannot draw exact conclusions on the role of well-defined brain regions. Although the RSN-level analysis might provide more robust insights on large-scale brain regional activity, our reported results will need to be confirmed with more appropriate analytical approaches such as source activity reconstruction by utilizing neuroimaging with precise anatomical localization (e.g., functional magnetic resonance imaging). The specificity of the identified neural correlates remains another open question in terms of SZ subtypes or neuropsychiatric conditions with an overlapping symptom profile (e.g., bipolar disorder or autism spectrum disorder). Even though during the correlation analyses we regressed out CPZ, our SZ population was heterogenous in terms of medication. Different pharmaceuticals commonly used in SZ treatment can affect EEG spectral properties in varying ways^79–81^, which in turn might had have an influence on the outcomes. According to recent synthesis, it is yet largely unknown how antipsychotic treatment might affect spectral characteristics linked to the E/I ratio^81^, and therefore while we cannot completely rule out that pharmacotherapy had an influence on the observed EEG alterations in SZ, this is an important research question that we intend to pursue in the future. Also, SZ disease duration varied in a broad range (from 0 to 28 years). It has been shown that early-stage and chronic SZ can exhibit different EEG signatures^82^, which likely introduced additional variability in our results. From the cross-sectional, resting-state design of this study it also follows that our biological understanding of EEG-symptom correlations remains elusive. Even though $\sigma ({\beta }_{{hi}})$ was meaningful in revealing between-group differences, we did not test explicitly if this truly indicates the presence – or in case of SZ, the loss of – multifractal dynamics in neural activity (e.g., as in refs. ^48,83,84^). This remains an open question but appears as a research avenue worth exploring in the future. Finally, results regarding the E/I ratio are commonly linked to glutamatergic/GABA-ergic activity, however altered functioning of the dopaminergic system is also evident in SZ^85^. In fact, recent notions imply the joint affectedness of dopamine-, GABA- and glutamatergic systems resulting in E/I imbalance in SZ^86^. Previous literature also reported on the effects of dopaminergic activity and medication on $1/{f}^{\beta }$ brain dynamics^87^ and thus sfuture efforts should make an attempt in synthesizing similar analysis outcomes with the dopamine hypothesis in SZ^16^.

In summary, we identified diminished fluctuations in high-frequency spectral in SZ when compared to HC, demonstrating that short-term temporal variability of neural E/I ratio can be distinctive and associated with clinical symptoms even if the baseline of E/I balance is maintained over longer time scales. Additionally, we addressed key methodological issues when inferring E/I ratio from the slope of EEG power spectra, confirming the relevance of assessing the power spectrum in a bimodal manner. Even though the work presented here can be considered first of its kind and thus exploratory in some regards, it provides novel and valuable insights into the neural signatures of SZ and raises questions hopefully facilitating future research in the field.

## Supplementary information

Supplementary Material

## Data Availability

Anonymized data (including raw EEG recordings, demographic and clinical information) generated throughout this study is available in a public Zenodo repository titled “Resting-state EEG, clinical, and demographics data from schizophrenia patients and age-matched healthy controls” at https://zenodo.org/records/14808296 (10.5281/zenodo.14808295).

## Code availability

All data analyses were carried out via Matlab (version 2023b) utilizing the EEGLAB toolbox^38^ (version 2020.0) along with custom analysis codes. Matlab codes developed and used for analyzing the data and visualizing outcomes (along with pre-processed EEG) are provided in a public GitHub repository at https://github.com/samuelracz/schizophrenia_EI_variability.
