# Supplementary material for: Reduced temporal variability of cortical excitation/inhibition ratio in schizophrenia
Source: Schizophrenia (Heidelb). 2025 Feb 18;11(1):20. doi: 10.1038/s41537-025-00568-3 (PMC11836122; doi:10.1038/s41537-025-00568-3)
Supplement: Supplementary file 1 — Supplementary Material [file 41537_2025_568_MOESM1_ESM.docx]

Supplementary material: Reduced temporal variability of cortical excitation/inhibition ratio in Schizophrenia

Frigyes Samuel Racz, Kinga Farkas, Melinda Becske, Hajnalka Molnar, Zsuzsanna Fodor, Peter Mukli and Gabor Csukly

# Irregular-resampling auto-spectral analysis (IRASA)

The purpose of IRASA is to decompose the power spectrum into a broadband fractal and superimposed oscillatory components [1]. IRASA directly builds on the coarse-graining spectral analysis (CGSA) introduced by Yamamoto and Hughson [2, 3] and exploits the same property of fractal processes. Briefly, fractal signals with a $1/f^{\beta}$-type spectrum are invariant under affine transformations [4], meaning that a resampled version of a signal will have a spectrum equal in distribution to that of the original, rescaled appropriately using its Hurst-exponent ($H$). Note that there is an exact correspondence between $H$ and $\beta$, and therefore they in fact capture the same property [2, 5]. Precisely, for given resampling factor $h$, the obtaining the Fourier transform of a fractal process resampled by $h$ ($F_{h}\left( \omega\right)$) relates to the original spectrum $F\left( \omega\right)$ according to

| $F_{h}\left( \omega\right)\triangleq h^{H}F\left( \omega\right)$, | (1) |
| --- | --- |

where $\omega$ denotes the angular frequency [1, 2]. In terms of the spectrum, this power-law dependency of power on frequency can be expressed as

| $\left\vert F(\omega) \right\vert^{2}\propto c\times\omega^{-\beta}$, | (2) |
| --- | --- |

where $c$ is a constant and $\beta$ is the fractal scaling exponent [6, 7]. This equation implies that fractal signals have non-zero power throughout the entire spectrum, which does not hold for oscillatory signals that only have non-zero power at the harmonic frequency $\omega_{0}$. From this and Equation (1) it follows that up- and down-sampling will produce a rescaled versions of the power spectrum for fractal signals, while it will result in a ‘re-location’ of spectral peaks in terms of oscillatory signals [1, 3].

IRASA assumes an additive model for neural signals ($y(t)$) with a fractal ($f(t)$) and an oscillatory ($x(t)$) component according to [1]:

| $y\left( t \right)=f\left( t \right)+x(t)$. | (3) |
| --- | --- |

According to the linearity property, the Fourier transform $Y(t)$ of $y(t)$ yields

| $Y\left( \omega\right)=F\left( \omega\right)e^{-j\gamma\left( \omega\right)}+X(\omega)e^{-j\varphi(\omega)}$, | (4) |
| --- | --- |

where $F(\omega)$ and $\gamma(\omega)$ denote the amplitude and phase of the fractal component at $\omega$, while $X(\omega)$ and $\varphi(\omega)$ are understood equivalently for the oscillatory component, and $j$ is the imaginary unit. The Fourier transforms of resampled versions of $y(t)$ – namely, $Y_{h}(\omega)$ when resampled by $h$ and $Y_{1/h}(\omega)$ when resampled by $1/h$ s – are obtained similarly. By utilizing Equation (1), it can be shown that the power spectra $S_{h}(\omega)$ and $S_{1/h}(\omega)$ of the rescaled time series are computed as [1, 7]

| $S_{h,h}\left( \omega\right)=Y_{h}\left( \omega\right)*\bar{Y_{h}\left( \omega\right)}=h^{2H}F^{2}\left( \omega\right)*\left\Vert A(\omega) \right\Vert^{2}$ | (5) |
| --- | --- |

and

| $S_{1/h,1/h}\left( \omega\right)=Y_{1/h}\left( \omega\right)*\bar{Y_{1/h}\left( \omega\right)}=h^{-2H}F^{2}\left( \omega\right)*\left\Vert B(\omega) \right\Vert^{2}$ | (6) |
| --- | --- |

where $A(\omega)$ and $B(\omega)$ are terms expressing the relationship between fractal and oscillatory components in terms of the ratio of their amplitudes and difference in their phases (for more details, please see [1]), and the horizontal bar denotes the complex conjugate. Finally, by computing the geometric mean of the up- and down-sampled power spectra, one obtains

| ${SS}_{h}\left( \omega\right)=\sqrt{h^{2H}F^{2}\left( \omega\right)*\left\Vert A\left( \omega\right) \right\Vert^{2}*h^{-2H}F^{2}\left( \omega\right)*\left\Vert B(\omega) \right\Vert^{2}}=F^{2}\left( \omega\right)\left\Vert A\left( \omega\right) \right\Vert\left\Vert B\left( \omega\right) \right\Vert.$ | (7) |
| --- | --- |

which is independent of $H$. Notably, assuming no phase relationship between fractal and oscillatory components, the part $\left\| A\left( \omega\right) \right\|\left\| B\left( \omega\right) \right\|$ is equal to 1 in all cases except when $\omega$ is equal to $\omega_{0}h$ or $\omega_{0}/h$ where $\omega_{0}$ is the harmonic frequency of $x(t)$, and therefore the power spectrum will contain oscillatory peaks superimposed on the broadband fractal component relocated from the original frequency $\omega_{0}$ by factors $h$ and $1/h$ [1]. Consequently, obtaining ${SS}_{h}\left( \omega\right)$ for multiple $h$ and $1/h$ pairs will yield a set of power spectra in which the fractal component is constant, while oscillatory peaks are found at different frequencies depending on $h$ (and $1/h$). Therefore, taking the median over these – that is robust against outliers caused by the oscillatory peaks – will produce an unbiased estimate of the fractal power spectrum. Finally, one can estimate the spectral slope (scaling exponent) $\beta$ from the fractal spectral component without the biasing effects of oscillatory peaks. Additionally, one can also obtain an isolated estimate for the oscillatory spectrum by simply subtracting the fractal spectrum from that of the original (i.e., not resampled) time series. The procedure outlined in Equations (3)-(7) is independent of $H$ (and thus $\beta$) and therefore can be applied to multimodal signals. Also, it is not limited to a single oscillatory component but works equivalently for an arbitrary number of peaks, as long as the number of different rescaling factors $h$ is large enough [1]. Using a large-enough set of non-integer rescaling factor pairs $h$ and $1/h$ also ensures that relocated oscillatory peaks do not overlap on case of multiple harmonic components.

# Supplementary Figures

#
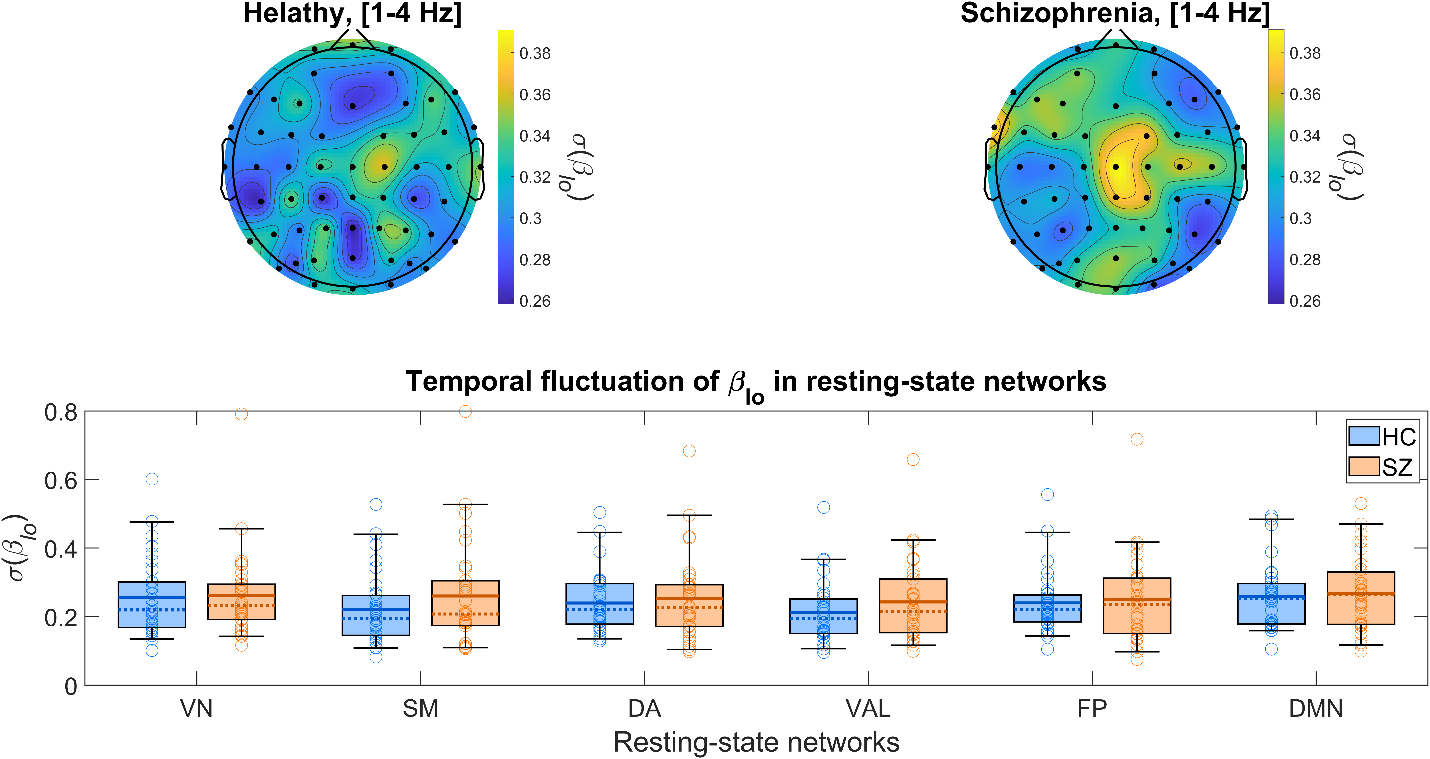


**Figure S1.** Temporal variability of $\beta_{lo}$ in the healthy (HC) and schizophrenia (SZ) groups. The upper left and right panels show the topology of $\sigma(\beta_{lo})$ in HC and SZ, respectively. The lower panel shows resting-state network (RSN)-wise analysis of $\sigma(\beta_{lo})$. In all box plots, continuous and dotted lines indicate the mean and median, respectively, the rectangular area denotes the inter-quartile range, horizontal whiskers whiskers indicate 5^th^ and 95^th^ percentile, and circles show the individual samples. Note that no statistically significant differences were found between HC and SZ. $\beta_{lo}$: low-range (1-4 Hz) spectral exponent; $\sigma(\beta_{lo})$: standard deviation of $\beta_{lo}$ over time.

# References

1. Wen, H.G. and Z.M. Liu, *Separating Fractal and Oscillatory Components in the Power Spectrum of Neurophysiological Signal.* Brain Topography, 2016. **29**(1): p. 13-26.

2. Yamamoto, Y. and R.L. Hughson, *Coarse-Graining Spectral-Analysis - New Method for Studying Heart-Rate-Variability.* Journal of Applied Physiology, 1991. **71**(3): p. 1143-1150.

3. Yamamoto, Y. and R.L. Hughson, *Extracting Fractal Components from Time-Series.* Physica D, 1993. **68**(2): p. 250-264.

4. Mandelbrot, B.B. and J.W. Van Ness, *Fractional Brownian motions, fractional noises and applications.* SIAM review, 1968. **10**(4): p. 422-437.

5. Eke, A., et al., *Physiological time series: distinguishing fractal noises from motions.* Pflugers Archiv : European journal of physiology, 2000. **439**(4): p. 403-415.

6. Eke, A., et al., *Fractal characterization of complexity in temporal physiological signals.* Physiological measurement, 2002. **23**(1): p. 1-38.

7. Racz, F.S., et al., *Multiple-Resampling Cross-Spectral Analysis: An Unbiased Tool for Estimating Fractal Connectivity With an Application to Neurophysiological Signals.* Frontiers in Physiology, 2022. **13**.
